# Comparison of EEG Signal Spectral Characteristics Obtained with Consumer- and Research-Grade Devices

**DOI:** 10.3390/s24248108

**Published:** 2024-12-19

**Authors:** Dmitry Mikhaylov, Muhammad Saeed, Mohamed Husain Alhosani, Yasser F. Al Wahedi

**Affiliations:** Abu Dhabi Maritime Academy, Abu Dhabi P.O. Box 54477, United Arab Emirates; muhammad.saeed@adports.ae (M.S.);

**Keywords:** EEG signal quality, consumer-grade EEG devices, wearable EEG technology, dry electrodes, spectral analysis, EEG device comparison

## Abstract

Electroencephalography (EEG) has emerged as a pivotal tool in both research and clinical practice due to its non-invasive nature, cost-effectiveness, and ability to provide real-time monitoring of brain activity. Wearable EEG technology opens new avenues for consumer applications, such as mental health monitoring, neurofeedback training, and brain–computer interfaces. However, there is still much to verify and re-examine regarding the functionality of these devices and the quality of the signal they capture, particularly as the field evolves rapidly. In this study, we recorded the resting-state brain activity of healthy volunteers via three consumer-grade EEG devices, namely PSBD Headband Pro, PSBD Headphones Lite, and Muse S Gen 2, and compared the spectral characteristics of the signal obtained with that recorded via the research-grade Brain Product amplifier (BP) with the mirroring montages. The results showed that all devices exhibited higher mean power in the low-frequency bands, which are characteristic of dry-electrode technology. PSBD Headband proved to match BP most precisely among the other examined devices. PSBD Headphones displayed a moderate correspondence with BP and signal quality issues in the central group of electrodes. Muse demonstrated the poorest signal quality, with extremely low alignment with BP. Overall, this study underscores the importance of considering device-specific design constraints and emphasizes the need for further validation to ensure the reliability and accuracy of wearable EEG devices.

## 1. Introduction

The electroencephalogram (EGG) is a non-invasive method to record brain activity, specifically aggregated post-synaptic potentials of large groups of neurons firing synchronously [1,2]. EGG possesses outstanding prospects for becoming a first-layer neuroimaging method due to its cost-efficiency and relative technological simplicity compared to other neuroimaging methods, such as functional MRI. It is widely used for neuroscientific research, clinical diagnostics, and brain–computer interface (BCI) construction [3,4]. While conventional research-grade EEG devices involve the usage of gel or other substances to improve skin–electrode conductivity and wired connection of electrodes with an amplifier, the technological simplicity of EEG allows the development of compact, wearable, and wireless devices based on dry-electrode technology. Dry electrodes make direct contact with the scalp, eliminating the need for gel or other substances to improve conductivity [5], while the quality of the signal they capture is still comparable with that of gel-based electrodes [6].

The popularity of wearable EEG devices with dry electrodes has been rapidly increasing in recent years. These devices offer several advantages, such as enabling out-of-lab brain activity monitoring, which opens new opportunities for academic and clinical research [7]. This is particularly beneficial for long-term studies and continuous patient monitoring. This provides more accurate and comprehensive data compared to brief laboratory sessions. Second, mounting such a device does not require the assistance of a trained EEG technician, which means that EEG is getting closer to ordinary users, and they may benefit from EEG-based neurofeedback training, mental health monitoring, and BCI [3,8,9]. An additional factor that further brings such EEG-based devices closer to real-life applications is their affordable cost, and they may be used widely to enhance the well-being and quality of life of people with psychiatric diseases and neurological disabilities as well as those of neurotypical people. Taken together, advancements in this field are highly sought after.

However, there is still much to validate and double-check regarding how these devices actually work and the qualities of the signal they capture, especially now, when the field is rapidly developing [10,11]. Due to technical differences from research-grade EEG devices with gel-based electrodes, the signal captured by the low-density wearable ones with dry electrodes cannot be the same. For example, the electrodes’ design implies higher impedance levels and proneness to artifacts [12], and the placement of electrodes, especially the ground and reference, also has a huge impact on the quality of the signal and applicability of a device for particular needs [13,14].

The most frequent non-clinical application of wireless EEG devices is in cognitive state monitoring, such as for concentration, meditative states, drowsiness, etc. [14,15,16,17]. This monitoring is usually based on spectral analysis of the EEG rhythms recorded during the resting state. Thus, following previous studies on consumer-grade EEG device validation [18], we focused our analysis on the most practically needed metrics based on spectral power measures in different frequency bands, such as the most stable one—Berger’s effect, suppression of the alpha rhythm power while the eyes are open [19]—as well as the mean power of the delta, theta, alpha, beta, and gamma rhythms.

In the present study, we evaluated the spectral characteristics of the signal obtained via low-density wearable systems, such as PSBD Headband Pro (PSBD LLC, Abu Dhabi, United Arab Emirates), PSBD Headphones Lite (PSBD LLC, Abu Dhabi, United Arab Emirates), and Muse S Gen 2 (InterAxon LLC, Toronto, ON, Canada), and compared them with that obtained via the state-of-the-art research-grade system, the Brain Product ActiChamp Plus amplifier (BP) (Brain Products GmbH, Munich, Germany), with the mirroring montages.

PSBD Headband and Headphones were previously validated only once in relation to the NVX EEG system (MCS, Mosco, Russia), and the results showed a fine alignment of the PSBD device’s spectral characteristics with the NVX in general. However, given some limitations of a previous study and the usage of a different research-grade device, we consider it valuable to retest the findings [13,14]. At the same time, Muse is a much more widely used device for now. There are many publications upon its validation, and their results are rather controversial. Some studies focused more on the spectral characteristics of the signal obtained via Muse point out its high susceptibility to artifacts and test–retest variability [20,21], but there is evidence that Muse performs reliably in ERP research [16].

Distinct types of electrodes are used in Muse and PSBD devices. Muse employs flat electrodes covered with conductive silver ink, while PSBD devices employ multi-pin electrodes, which possess more stable contact and lower impedance, although they are less comfortable during long sessions of recording [22]. Nonetheless, we expected more or less similar signal quality of these devices in comparison to BP, with all devices exhibiting weaker signal quality in low frequencies, which is characteristic of the signal obtained via dry electrodes [23].

To sum up, the current study aimed to validate and compare three wearable dry-electrode EEG devices versus the finest investigation-level amplifier in terms of the spectral characteristics of the signal.

## 2. Methods

The study sample comprised 19 participants (9 females, age 24 ± 10.3). All participants had no history of psychiatric or neurological diseases or traumatic brain injuries. The participants were instructed to have proper sleep the night before the experiment (at least 8 h), not to consume alcohol the day before, not to drink coffee or energy drinks in the morning prior to the experiment, and not to smoke for 1 h before the study. Also, the participants were asked not to use hair conditioners or apply cosmetics on their foreheads.

The participants received a fixed compensation of USD 25 for their participation and signed the informed consent form before the start of the experiment. This study was conducted in accordance with the Helsinki Declaration and was approved by the ethical committee.

According to the power analysis conducted in G×power [24], the sample of 13 participants would be enough to detect a significantly strong correlation (0.7), with 0.05 alpha error probability and 80% power.

### 2.1. Data Acquisition and Experimental Procedure

The EEG recordings were obtained using 4 devices—PSBD Headband Pro (PSBD-band), PSBD Headphones Lite (PSBD-headphones), Muse S Gen 2 (Muse), and a Brain Product (BP) amplifier with ActiChamp Plus—in 2 montages.

The devices’ montages and specification for mirror-montage BP recordings are summarized in Table 1. Muse has 4 dry electrodes located analogous to AF7, AF8, TP9, and TP10 with FPz used as a reference and FP1, FP2 as a ground electrode. PSBD band has 4 dry electrodes (T3, T4, O1, O2), with FPz as a reference and FP1, FP2 as a ground electrode, similar to Muse. PSBD-headphones also has 4 recording channels (C3, C4, A1, A2), Cz as a reference, and FT9 as a ground electrode. Each electrode of PSBD band and PSBD-headphones contains 25 pogo pins. There were 2 montage versions of BP recordings: BP-band, with 6 recording channels (O1, O2, T7, T8, F7, F8), FPz as a reference, and FP1 as a ground electrode, which was used for comparison with PSBD band and Muse; and BP-headphones, with 4 recording electrodes (C3, C4, TP9, TP10), Cz as a reference, and FT9 as a ground electrode, which was used for comparison with PSBD-headphones.

The corresponding electrodes for the BP recordings were chosen based on the real electrodes’ position in relation to BP 10-10 montage. Therefore, T3 and T4 of PSBD Headband and TP9 and TP10 of Muse correspond to T7 and T8 of the BP montage. AF7 and AF8 of Muse correspond to F7 and F8 of BP. A1 and A2 of PSBD-headphones correspond to TP9 and TP10 of BP.

It took 15 min to allocate each device. The recordings started after a satisfactory signal quality was obtained. For BP, the impedance levels were kept below 30 k Ohm. For the PSBD devices, the impedance levels were kept below 300 k Ohm. The Muse device does not have direct access to impedance values, but the indicators available in the Muse software were kept “green”.

There were two conditions for obtaining the EEG recordings: eyes closed and open for 3 min each. First, the recordings were obtained via the dry-electrode devices in a random order. This was followed by EEG recording via the BP amplifier, first with the montage mirroring the PSBD-headphones and Muse montages, then with the montage mirroring the PSBD band montage.

### 2.2. EEG Data Analysis

The EEG data analysis was conducted in MNE Python [25]. As our motivation was to compare the quality of the raw signals obtained with different devices, the preprocessing included only the filtration with an FIR filter in the range between 0.5 and 40 Hz. The Muse recordings were resampled from 256 Hz to 250 Hz to obtain equal sampling frequency among all the devices.

Pairs of electrodes at the available sites (frontal, temporal, central, and occipital) were combined. The spectral analysis was conducted with Welch’s method (Welch, 1967) (Hanning window function, time window of 2 s with ½ overlap, frequency step of 0.5 Hz). The resulting power spectral density (PSD) values were converted to the dB scale (10 × log10(PSD)) and averaged over the frequency ranges of interest, namely the delta (1–3.5 Hz), theta (4–7.5 Hz), alpha (8–13 Hz), low beta (13.5–20 Hz), high beta (20.5–30 Hz), and gamma (30.5–40 Hz) bands.

### 2.3. Statistical Analysis

The statistical analysis was conducted separately for each site—frontal (available only in Muse and BP-band), central (available in PSBD-headphones and BP-headphones), occipital (PSBD band and BP-band), and temporal (all devices)—and frequency band (delta, theta, alpha, low beta, high beta, and gamma).

In order to compare spectral characteristics of signals obtained with the devices, we used repeated measures ANOVA with the factors of device, condition, and their interaction. The Greenhouse–Geisser correction was applied in case of violation of the sphericity assumption. Tukey’s HSD was used for the post hoc analysis.

In addition, we conducted a correlational analysis to assess the correspondence of the spectral power measures obtained with Muse, PSBD-band, and PSBD-headphones with those obtained via their mirror-montage version of BP. The analysis was conducted separately for each specific frequency band.

## 3. Results

### 3.1. Signal Quality Evaluation

The majority of the Muse recordings (N = 17) exhibited poor signal quality at the TP9 and TP10 channels (temporal site). The signal had no properties of EEG and was non-physiological; a screenshot of such a recording. The exact reason for this result is unknown, as the available indicators of the signal quality indicated a satisfactory signal quality (the green indicators in the Muse recording software are the only available option to assess impedance levels). At the same time, the signal on the frontal electrodes was satisfactory. Therefore, we excluded the Muse temporal site from further analysis and analyzed the signal only from the frontal site.

The signal obtained with all other devices was satisfactory.

### 3.2. Power-Spectrum Plots

**Figure 1:**
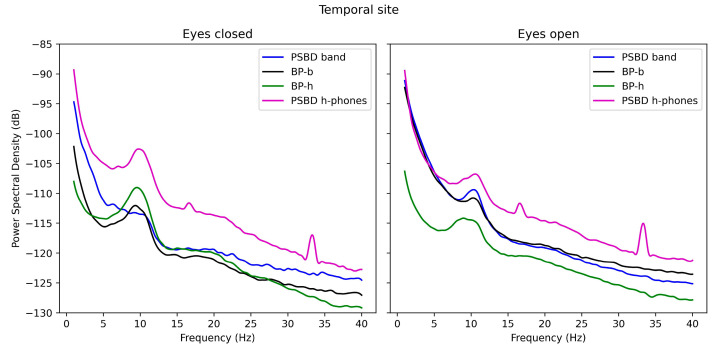
The PSD plots for the signals obtained via PSBD-band, BP-band (BP-b), BP-headphones (BP-h), and PSBD-headphones (PSBD h-phones) in the open- and closed-eye conditions.

The spectral analysis was conducted separately for each location site. In Figure 1, the spectral functions of the signals obtained via PSBD-band, PSBD-headphones, BP-band, and BP-headphones at the temporal site are presented. PSBD-headphones exhibited the highest overall power, with spikes at approximately 16 and 34 Hz. The rise in power in the alpha rhythm in the closed-eye condition was noticeable for each device but was the smallest in the case of the PSBD-band.

**Figure 2:**
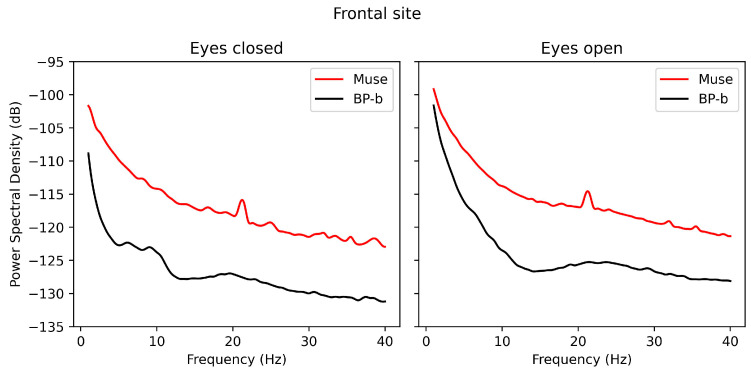
The PSD plots for the signals obtained via Muse and BP-band (BP-b) in the open- and closed-eye conditions.

In Figure 2, the plot represents the PSD of the signals obtained via Muse and BP-band at the frontal site. A weak correspondence of the signal PSD between the devices can be noted, with Muse exhibiting overall higher power and a spike at 20–22 Hz. While a slight elevation in alpha power in the closed-eye condition can be seen for the BP-band, it is absent on the Muse plot.

**Figure 3:**
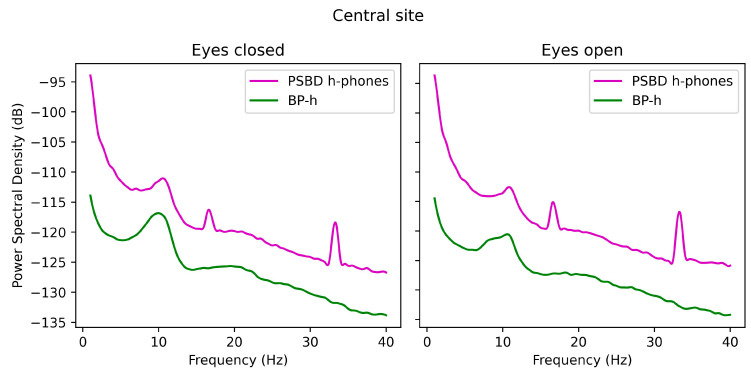
The PSD plots for the signals obtained via PSBD-headphones and BP-headphones (BP-h) in the open- and closed-eye conditions.

In Figure 3, the PSD plots for the signals obtained via PSBD-headphones and BP-headphones at the central site are presented. Similarly to the temporal site, PSBD-headphones possessed overall higher power and spikes at approximately 16 and 34 Hz.

**Figure 4:**
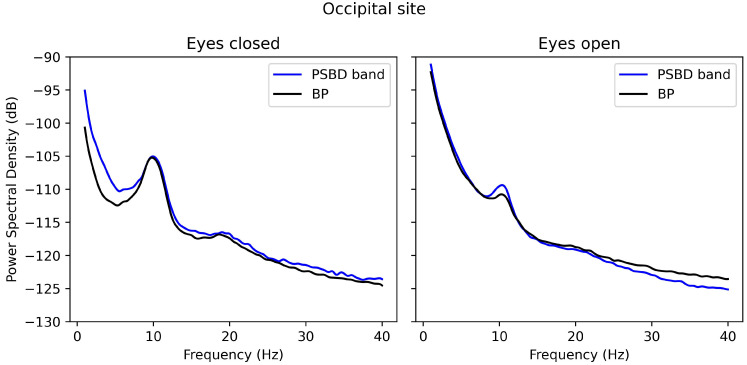
The PSD plots for the signals obtained via PSBD band and BP-band (BP) in the open- and closed-eye conditions.

Figure 4 represents the PSD plots of the signals obtained with PSBD band and BP-band at the occipital site. The plots are exceptionally similar except for slight divergences, such as those in the delta and theta bands in the closed-eye condition.

### 3.3. ANOVA of the Power in Specific Frequency Bands

#### 3.3.1. Frontal Site: Muse and BP-Band

**Figure 5:**
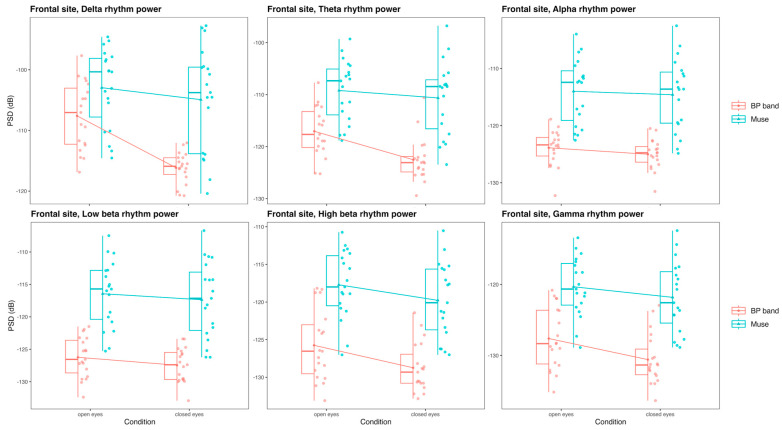
Box plots illustrating differences in the PSD values for the delta, theta, alpha, low beta, high beta, and gamma rhythms obtained with Muse and BP-band in the closed-/open-eye conditions at the frontal site.

At the frontal site, there were differences between the spectral powers obtained with Muse and BP-band in all frequency bands. The visualization of PSD values for each frequency band, depending on the device and condition, is provided in Figure 5.

In the delta band, the results of ANOVA showed significant effects of device [F(1, 18) = 36.33, *p* < 0.001], condition [F(1, 18) = 18.42, *p* < 0.001], and their interaction [F(1, 18) = 7.10, *p* = 0.016]. The effect of the condition may point to the fact that such a position of the reference electrode made the signal prone to eye-movement and blink artifact contamination, which was practically absent in the closed-eye condition. While the relative absence of eye movements and blinks stabilized the BP-band signal, it did not work the same for Muse. The difference between the devices was more pronounced in the closed-eye condition (T(18) = −5.53, *p* < 0.001) than in the open-eye condition (T(18) = −3, *p* = 0.008). Overall, the delta power values were higher for Muse (T(18) = −6.03, *p* < 0.001).

In the theta band, the results were practically similar. ANOVA showed significant effects of device [F(1, 18) = 43.52, *p* < 0.001], condition [F(1, 18) = 11.3, *p* = 0.003], and their interaction [F(1, 18) = 4.79, *p* = 0.042]. The difference between the devices was slightly more pronounced in the closed-eye condition (T(18) = −5.9, *p* < 0.001) than in the open-eye condition (T(18) = −5.3, *p* < 0.001).

In the alpha band, there was only a significant effect of the device [F(1, 18) = 50.6, *p* < 0.001], with Muse exhibiting overall higher alpha rhythm power (T(18) = −7.1, *p* < 0.001). In general, the absence of the condition effect may point to the absence or weakness of functional properties of the alpha rhythm at the frontal site.

Similar results were observed for the low beta-band, with only a significant effect of the device being present [F(1, 18) = 60.3, *p* < 0.001], and Muse exhibiting higher power values (T(18) = −7.8, *p* < 0.001). High beta power values depended on the condition [F(1, 18) = 18.62, *p* < 0.001] and device factors [F(1, 18) = 71.22, *p* < 0.001], with Muse also exhibiting higher power values (T(18) = −8.4, *p* < 0.001).

The gamma band power was also significantly different in devices [F(1, 18) = 76.93, *p* < 0.001] and conditions [F(1, 18) = 11.85, *p* = 0.003]. Muse exhibited higher power (T(18) = −8.8, *p* < 0.001).

#### 3.3.2. Temporal Site: PSBD-Band, BP-Band, PSBD-Headphones, and BP-Headphones

**Figure 6:**
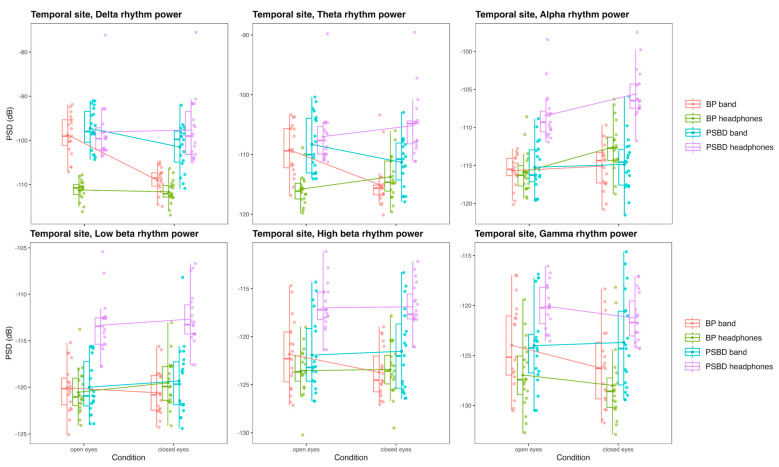
Box plots illustrating differences in the PSD values for the delta, theta, alpha, low beta, high beta, and gamma rhythms obtained with PSBD-band, BP-band, PSBD-headphones, and BP-headphones in the closed-/open-eye conditions at the temporal site.

The temporal site was presented for all devices, although Muse was excluded from analysis of this site due to excessive artifacts. The comparison of the powers of the delta, theta, alpha, low beta, high beta, and gamma rhythms obtained with PSBD-band, BP-band, PSBD-headphones, and BP-headphones in the closed-/open-eye conditions is visualized in Figure 6.

Power in the delta band was different depending on the device [F(1.66, 29.85) = 40.21, *p* < 0.001], the condition [F(1, 18) = 34.86, *p* < 0.001], and their interaction [F(2.4, 43.27) = 39.3, *p* < 0.001]. Overall, there were no significant differences between PSBD band and PSBD-headphones; PSBD-headphones exhibited higher power than BP-headphones (T(18) = −8.28, *p* < 0.001), and the same was true for BP-band and PSBD band (T(18) = −5.7, *p* < 0.001). The fact that the BP-band exhibited higher power than BP-headphones, especially in the open-eye condition, may illustrate the influence of the reference electrode placement (T(18) = 10.86, *p* < 0.001).

In the theta band, the results were practically similar, with significant effects of the device [F(1.98, 35.57) = 25.54, *p* < 0.001], the condition [F(1, 18) = 5.36, *p* = 0.033], and their interaction [F(2.25, 40.56) = 23.98, *p* < 0.001]. Overall, PSBD devices’ power still did not differ significantly, although the PSBD-headphones yielded higher values specifically in the closed-eye condition (T(18) = −4.3, *p* = 0.002). BP-band exhibited significantly higher power than BP-headphones in general (T(18) = 4.02, *p* = 0.041) and specifically in the open-eye condition (T(18) = 6.07, *p* < 0.001), in which eye-movement artifacts were much more prevalent. Both PSBD devices exhibited higher powers than their BP versions (BP-band vs. PSBD-band: T(18) = −3.8, *p* = 0.007; BP-headphones vs. PSBD-headphones: T(18) = −7.69, *p* < 0.001).

In the alpha band, there were significant effects of the device [F(2.31, 41.58) = 126.46, *p* < 0.001], the condition [F(1, 18) = 17.94, *p* < 0.001], and their interaction [F(2.25, 40.57) = 10.7, *p* < 0.001]. The only significant differences between the devices observed were for BP-headphones and PSBD-headphones (T(18) = −13.6, *p* < 0.001) and PSBD band and PSBD-headphones (T(18) = −8.03, *p* < 0.001). Alpha rhythm power values obtained with BP-band and PSBD band did not differ. Also, there was no significant difference between BP-band and BP-headphones in this frequency band in general, though in the closed-eye condition, a difference existed (T(18) = −6.08, *p* < 0.001). Interestingly, there was no significant Berger’s effect for BP-band and PSBD-band, although for PSBD-headphones (open vs. closed: T(18) = −5.3, *p* < 0.001) and BP-headphones (open vs. closed: T(18) = −6.57, *p* < 0.001) it was obvious.

The low beta rhythm power was modulated only by the factor of the device [F(2.48, 44.71) = 64.17, *p* < 0.001], with PSBD-headphones exhibiting the highest power values in comparison to all other devices (BP-headphones vs. PSBD-headphones: T(18) = −12.6, *p* < 0.001 and PSBD band vs. PSBD-headphones: T(18) = −8.9, *p* < 0.001). The same was applicable to the high beta rhythm power, with the factor of the device being the only significant one [F(2.5, 44.94) = 33.9, *p* < 0.001], and PSBD-headphones exhibited the highest power values (BP-headphones vs. PSBD-headphones: T(18) = −11.87, *p* < 0.001 and PSBD band vs. PSBD-headphones: T(18) = −6.35, *p* < 0.001).

In the gamma band, there were significant effects of the device [F(2.54, 45.72) = 18.96, *p* < 0.001] and condition [F(1, 18) = 6.51, *p* = 0.02]. Bp-band and PSBD band did not differ in gamma power. The values obtained with PSBD-headphones were higher than those of BP-headphones (T(18) = −8.52, *p* < 0.001) and BP-band (T(18) = −3.58, *p* = 0.01). Also, BP-band power values were higher than those of BP-headphones (T(18) = 2.92, *p* = 0.04).

#### 3.3.3. Occipital Site: PSBD Band and BP-Band

**Figure 7:**
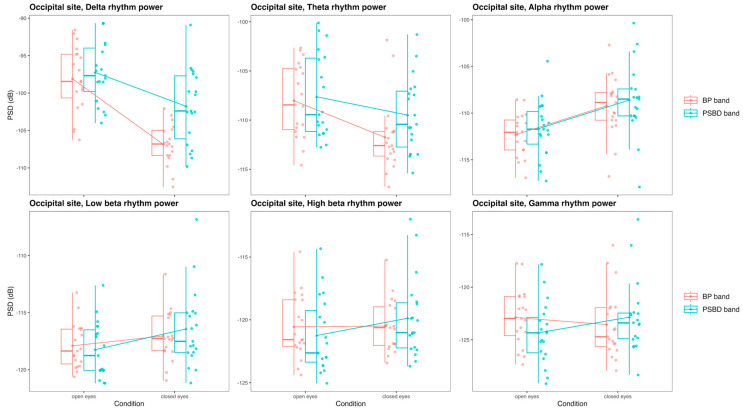
Box plots illustrating differences in the PSD values for the delta, theta, alpha, low beta, high beta, and gamma rhythms obtained with PSBD band and BP-band in the closed-/open-eye conditions at the occipital site.

The occipital site was presented for PSBD-headphones and BP-headphones, and the comparison of the mean power values obtained with these devices in the delta, theta, alpha, low beta, high beta, and gamma rhythms in the open-eye and closed-eye conditions is visualized in Figure 7.

In the delta band, there were significant effects of the device [F(1, 18) = 16.98, *p* < 0.001], the condition [F(1, 18) = 52.44, *p* < 0.001], and their interaction [F(1, 18) = 9.79, *p* = 0.006]. While with the condition factor eliminated, overall, PSBD band exhibited higher power values (T(18) = −4.12, *p* < 0.001), there was no significant difference in power in the open-eye condition specifically, and only in the closed-eye condition did it differ (T(18) = −4.14, *p* < 0.001). For both devices, power in the closed-eye condition was lower due to the relative absence of eye movements and blinks.

A similar situation was observed in the theta band. There were significant effects of device [F(1, 18) = 8.63, *p* = 0.009] and condition [F(1, 18) = 10.43, *p* = 0.005]. PSBD band exhibited higher power values (T(18) = −2.94, *p* = 0.009).

In the alpha band, the devices’ PSD values did not differ. The only significant factor was the condition factor [F(1, 18) = 25.05, *p* < 0.001]. The alpha suppression effect was significant for both PSBD band (open vs. closed: T(18) = −4.93, *p* < 0.001) and BP-band (open vs. closed: T(18) = −4.37, *p* < 0.001).

No statistically significant differences existed between the power values obtained with PSBD band and BP-band in the low beta, high beta, and gamma bands.

#### 3.3.4. Central Site: PSBD-Headphones and BP-Headphones

**Figure 8:**
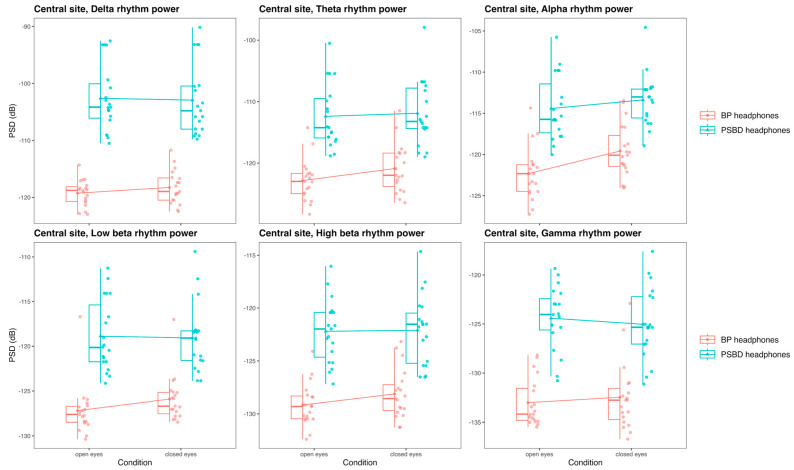
Box plots illustrating differences in the PSD values for the delta, theta, alpha, low beta, high beta, and gamma rhythms obtained with PSBD-headphones and BP-headphones in the closed-/open-eye conditions at the central site.

The central site was presented for PSBD-headphones and BP-headphones, and the comparison of the mean power values obtained with these devices in the delta, theta, alpha, low beta, high beta, and gamma rhythms in the open-eye and closed-eye conditions is visualized in Figure 8.

In the delta band, the only significant factor was the factor of the device [F(1, 18) = 103.06, *p* < 0.001], and PSBD-headphones exhibited higher power (T(18) = −10.15, *p* < 0.001). Theta band power depended on the device factor [F(1, 18) = 62.9, *p* < 0.001] with higher values for PSBD-headphones (T(18) = −7.93, *p* < 0.001), and the effects of condition [F(1, 18) = 3.76, *p* = 0.013] and interaction [F(1, 18) = 4.99, *p* = 0.038] were also significant. The difference between devices was more pronounced in the open-eye condition (T(18) = −8.12, *p* < 0.001) than in the closed-eye condition (T(18) = −7.2, *p* < 0.001).

In the alpha band, there were significant effects of device [F(1, 18) = 61.64, *p* < 0.001], condition [F(1, 18) = 21.23, *p* < 0.001], and their interaction [F(1, 18) = 9.5, *p* = 0.006]. The power values of PSBD-headphones were higher than those of BP-headphones (T(18) = −7.85, *p* < 0.001). The alpha rhythm suppression effect was significant for BP-headphones (open vs. closed: T(18) = −5.72, *p* < 0.001), while for PSBD-headphones it was weaker, on the level of tendency only (open vs. closed: T(18) = −2.04, *p* = 0.056).

In the low beta band, there were significant effects of device [F(1, 18) = 86.45, *p* < 0.001] and device–condition interaction [F(1, 18) = 5.25, *p* = 0.006]. Overall, PSBD-headphones’ power was higher (T(18) = −9.3, *p* < 0.001), and this distinction was slightly more pronounced in the open-eye condition (T(18) = −9.18, *p* < 0.001) than in the closed-eye one (T(18) = −8.04, *p* < 0.001).

In the high beta and gamma bands, the effects of the device were significant [beta: F(1, 18) = 88, *p* < 0.001; gamma: F(1, 18) = 65.77, *p* < 0.001], and PSBD-headphones exhibited higher power values (high beta: T(18) = −9.38, *p* < 0.001; gamma T(18) = −8.1, *p* < 0.001).

### 3.4. Correlational Analysis

For the correlational analysis, the data points for the closed- and open-eye conditions were combined in one pool that resulted in N = 38 data points.

#### 3.4.1. Muse and BP-Band

**Figure 9:**
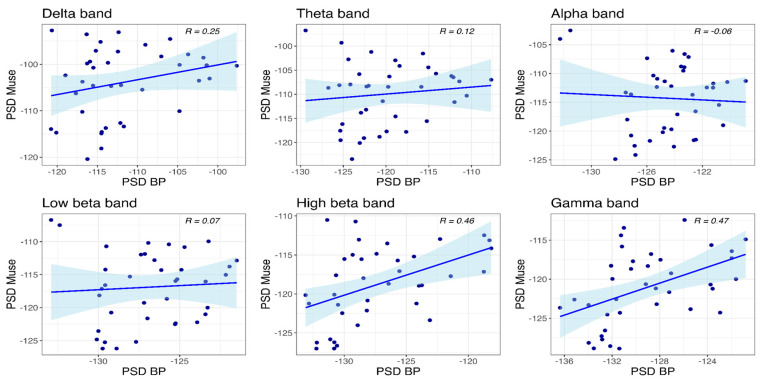
Scatter plots depicting power values obtained with Muse and BP-band at the frontal site.

The correlations for the frontal site are presented in Figure 9. Significant correlations between the power values of Muse and BP-band were observed only in the high beta (R(36) = 0.46, *p* = 0.004) and gamma bands (R(36) = 0.47, *p* = 0.003).

#### 3.4.2. PSBD Band and BP-Band

**Figure 10:**
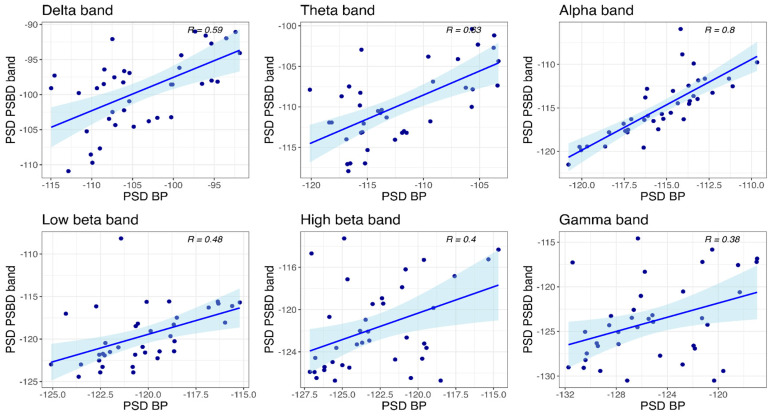
Scatter plots depicting power values obtained with PSBD band and BP-band at the temporal site.

Correlations between the power values obtained with PSBD band and BP-band at the temporal site are visualized in Figure 10. Significant correlations of the power values were found in all frequency bands: delta (R(36) = 0.59, *p* < 0.001), theta (R(36) = 0.63, *p* < 0.001), alpha (R(36) = 0.8, *p* < 0.001), low beta (R(36) = 0.48, *p* = 0.003), high beta (R(36) = 0.4, *p* = 0.012), and gamma (R(36) = 0.38, *p* = 0.019).

**Figure 11:**
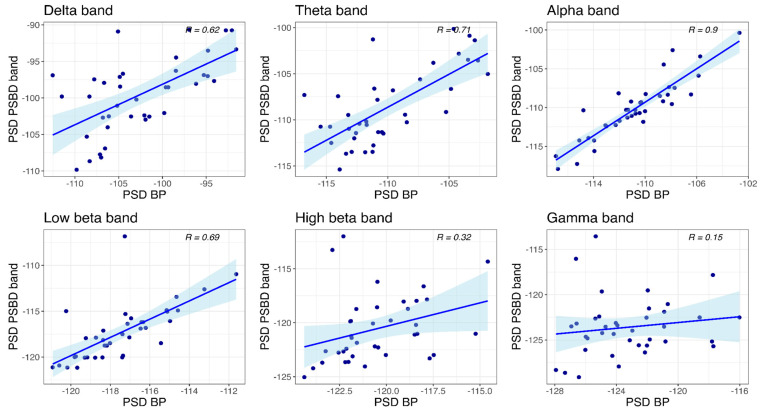
Scatter plots depicting power values obtained with PSBD band and BP-band at the occipital site.

At the occipital site (Figure 11), significant correlations between the power values obtained with PSBD band and BP-band were observed in all frequency bands except for the gamma band: delta (R(36) = 0.62, *p* < 0.001), theta (R(36) = 0.71, *p* < 0.001), alpha (R(36) = 0.9, *p* < 0.001), low beta (R(36) = 0.69, *p* < 0.001), and high beta (R(36) = 0.32, *p* = 0.049).

#### 3.4.3. PSBD-Headphones and BP-Headphones

**Figure 12:**
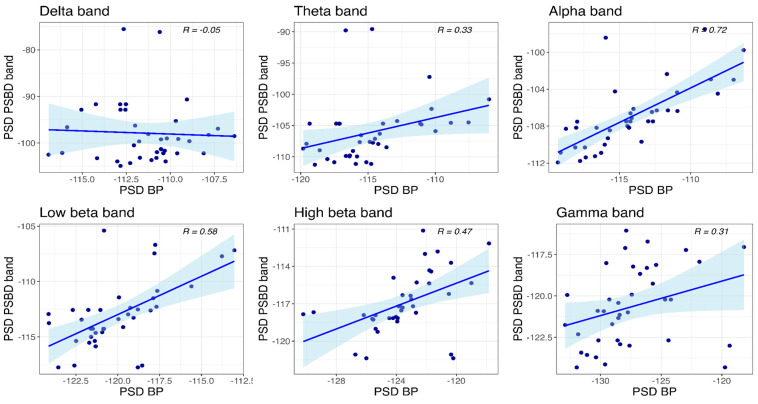
Scatter plots depicting power values obtained with PSBD headphones and BP-headphones at the temporal site.

Correlations between the power values obtained with PSBD-headphones and BP-headphones at the temporal site are visualized in Figure 12. Significant correlations between the mean PSD values were found in all frequency bands except for the delta and gamma bands: theta (R(36) = 0.33, *p* = 0.043), alpha (R(36) = 0.72, *p* < 0.001), low beta (R(36) = 0.58, *p* < 0.001), and high beta (R(36) = 0.47, *p* = 0.003).

**Figure 13:**
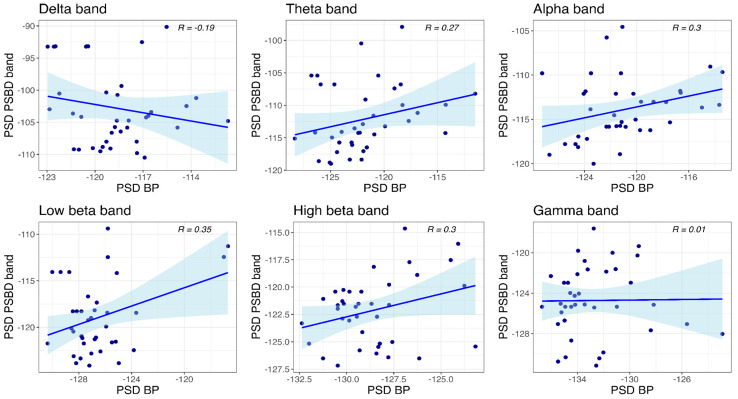
Scatter plots depicting power values obtained with PSBD headphones and BP-headphones at the central site.

At the central site (Figure 13), a significant correlation was observed only in the low beta band (R(36) = 0.35, *p* = 0.029).

## 4. Discussion

In the present study, we compared three wearable EEG devices with dry electrodes, namely PSBD Headband Pro (PSBD-band), PSBD Headphones Lite (PSBD-headphones), and Muse S Gen 2 (Muse), with the finest investigation-level Brain Product (BP) amplifier with gel-based electrodes in terms of spectral characteristics of the EEG signal in the resting state. The mirror-montages of BP were applied for a direct comparison with each wearable device, and this is the first precedent for such a direct comparison, which is particularly needed because ordinary people have started to use these devices more widely and rely on them as a tool to enhance their well-being [11].

As expected, the spectral characteristics of the signals obtained with consumer-grade devices were not perfectly aligned with those obtained via BP. Due to electrodes’ design constraints, consumer-grade devices exhibited higher noise levels and more proneness to artifacts [12]. Overall, the results demonstrate that the PSBD band exhibits the finest alignment with its mirror-montage BP recording. PSBD-headphones shows slightly weaker correspondence with the BP recording. The weakest alignment with BP is exhibited by Muse. However, details and nuances are to be considered.

It was previously shown that Muse signal quality is disturbed by muscle tension and eye-movement artifacts enhanced by the reference electrode placed in the frontal region with an overall broadband increase in signal power and that it has high test–retest variability [20,21]. In the study by [21], researchers also analyzed only the signal at the frontal site (one channel: Fp1). In our study, we observed very poor signal quality in the temporal channels and did not include them in further analysis and statistics, while the built-in indicators of signal quality were “green”, which denotes satisfactory impedance levels. At the same time, the signal at the frontal site showed significant differences from that of the BP-band (similar montage version of BP) in all frequency bands. In addition, there were no significant correlations in power values in the frequency bands lower than high beta, though the correlations were still moderate for the high beta and gamma bands.

Considering that Muse aims to provide information to guide meditation and relaxation sessions, our results raise many concerns. The first one is the obviously weak alignment, if any, with the research-grade EEG amplifier. The second concern is not related to Muse specifically but rather to the choice of frontal electrodes for meditation guiding devices. There were no alpha rhythm suppression effects at the frontal site for either Muse or BP-band, while the alpha rhythm is a crucial marker of meditative states. In a recent mindfulness study, no link was found between EEG metrics of meditation success provided by Muse and self-reported measures of the distress level and mindfulness score after one month of training [10].

At the same time, in previous studies, alpha rhythm asymmetries and peak frequencies were analyzed in the frontal channels of Muse [26]. What is important to note is that in this study, researchers applied some water to dry electrodes to decrease impedance and increase signal quality and re-referenced frontal channels to temporal ones. These practices are not implemented in the pipelines for customers and thus blur the real state of things. With these procedures applied, they reached significant correlations with a research-grade amplifier in all bands except for the gamma, which contradicts the results we obtained. In other studies, significant correlations of mean power were demonstrated and totally unsatisfactory signal quality in the temporal channels with a research-grade amplifier in all frequency bands [13] and the high quality of ERPs compatible with BP [16] were observed. With these points taken together, the signal quality of Muse requires further clarification and validation.

PSBD-headphones exhibited moderate signal quality and alignment with BP measures. There were obvious artifactual spikes on the power-spectrum plots at approximately 16 and 34 Hz, and the mean power values in all frequency bands differed significantly from those of BP, which were much lower. While there are significant correlations with BP measures in all frequency bands except for the delta and gamma at the temporal site, reaching up to 0.7 for the alpha rhythm, the correlations at the central site were notably weak, with the only significant one in the high beta band. PSBD-headphones showed a significant Berger’s effect at the temporal site, as did BP-headphones, although PSBD-headphones demonstrated no effect at the central site. A previous study comparing PSBD-headphones with a research-grade EEG device found higher correlations [13]. While PSB- headphones is a promising device given that EEG is embedded in real working headphones and that the position of channels is beneficial for potential mu-rhythm-based applications related to sensorimotor systems [27,28], the device may still need some work to enhance signal quality.

PSBD band showed the strongest correlations of mean power values with BP. The closest alignment was observed at the occipital site, reaching up to 0.9 correlation in the alpha band, while weaker correlations were found in the high beta and gamma bands. Berger’s effect of alpha rhythm suppression was well defined in both PSBD band and BP-band at the occipital site and absent at the temporal site. Given the presence of significant correlations with BP recording in all available sites and frequency bands from delta to high beta, the results further validate the PSBD band as a reliable and promising consumer-grade EEG device. Our results are in line with those of a previous study with PSBD band that also showed high mean power correlations with a research-grade device [13]. Significant differences between PSBD band and BP-band mean power values at both the occipital and temporal sites were shown only for delta and theta rhythms, which is expected and typical for dry-electrode devices in general [23,29].

However, it should be noted that certain design constraints lead to the proneness of the PSBD band and Muse devices to eye-movement and blink artifacts observed in lower frequencies. Namely, the placement of the reference electrode causes these problems. The effect of the reference electrode placement was comprehensively illustrated in our study when we compared mean powers of the delta and theta rhythms obtained with the devices: the values obtained with BP in the band and headphones montages cease to differ so largely in the closed-eye condition, which is not contaminated with eye-movement and blink artifacts.

Therefore, we assume that the delta and theta rhythm assessment would be more reliable during calm states with eyes closed. In addition, users should be informed of a possible bias and instructed to use devices for delta- and theta-rhythm-based metric assessment only in appropriate settings, eliminating eye movements and blinks. PSBD band can be considered a valuable tool for cognitive state monitoring and meditation guidance if the above-mentioned constraints are accounted for.

We aimed to designate spectral characteristics of the signals obtained with the consumer-grade devices just as they are, without applying sophisticated preprocessing techniques. Nonetheless, the successful development of this field also depends on the elaboration of appropriate and effective online preprocessing techniques that could be embedded into devices to enhance signal quality, given particular design constraints [30].

Among this study’s limitations, we would like to mention two major points. First, we acknowledge the fact that the Muse device used in the experiment could be corrupted. Although such signal quality could also be caused by electrical interference, it is important to note that the surroundings during EEG acquisition were identical for all recordings obtained. The issue might be related to the fact that the embedded signal quality indicators were misleading; thus, that raises a concern: how can an ordinary user notice a problem? Second, test–retest reliability analysis is necessary to further validate the spectral characteristics of signals obtained with wearable EEG devices.

In this study, we compared Muse, PSBD-band, and PSBD-headphones with mirror-montage recordings from the research-grade BP amplifier, focusing on signal spectral characteristics. PSBD band proved to match BP most precisely among the examined devices. PSBD-headphones demonstrated limited capacities at the central site, although the signal quality at the temporal site was satisfactory. The Muse device exhibited the lowest signal quality, showing minimal correspondence with BP.

### 4.1. Device-Specific Differences and Contributing Factors

The observed differences between the consumer-grade EEG devices and the research-grade BP system can largely be attributed to several key factors, including electrode design, reference electrode placement, and signal processing algorithms.

#### 4.1.1. Electrode Design

Research-grade systems like the BP amplifier employ gel-based electrodes, which provide superior conductivity and lower impedance levels compared to the dry electrodes used in consumer-grade devices. In contrast:

- Muse uses flat electrodes coated with conductive silver ink, which are prone to signal degradation and may result in poor contact, particularly at temporal sites.
- PSBD devices use multi-pin dry electrodes, which improve contact stability and reduce impedance to some extent. However, the higher impedance compared to gel electrodes still leads to increased noise, especially in lower-frequency bands such as delta and theta.

#### 4.1.2. Reference Electrode Placement

The placement of reference electrodes plays a significant role in signal quality and artifact contamination:

- In Muse, the frontal placement of reference electrodes increases susceptibility to eye-movement and blink artifacts. This likely contributed to the poor signal quality observed at the temporal electrodes.
- In the PSBD Headphones, the central reference placement (Cz) is less prone to frontal artifacts but still shows some susceptibility to muscle-related noise at the central site.
- The PSBD Headband demonstrated better alignment with the BP system, particularly at the occipital site, where alpha suppression (Berger’s effect) was clearly observed. This highlights the importance of electrode and reference placement in mitigating artefacts and improving reliability.

#### 4.1.3. Signal Processing Algorithms

Differences in built-in signal processing pipelines among devices could also explain some of the discrepancies:

- Muse relies on proprietary algorithms for signal quality metrics, which may not always accurately reflect impedance or noise levels, as observed in our study.
- Research-grade BP systems employ robust preprocessing, filtering, and impedance monitoring techniques that significantly enhance signal fidelity.
- While the PSBD devices performed better than Muse, slight deviations in spectral power, particularly in the low-frequency bands, suggest opportunities for improving their signal processing algorithms to account for dry-electrode noise and artifacts.

#### 4.1.4. Artifact Susceptibility

Consumer-grade devices, by design, are intended for ease of use and portability. However, these benefits often come at the cost of increased artifact susceptibility due to factors like electrode type, signal preprocessing, and reference placement. For example:

- Temporal and central recordings with PSBD Headphones showed higher noise levels and artifact spikes at 16 Hz and 34 Hz, which are likely linked to external interference or device-specific design constraints.
- Signal quality in Muse’s temporal site was completely compromised, suggesting a need for better artifact handling algorithms or improved electrode placement.

## 5. Future Directions

The current study highlights significant differences between consumer-grade and research-grade EEG devices, underscoring the need for further investigation to enhance the performance and applicability of wearable EEG technologies [2,31]. To address the limitations and advance this field, several future research directions are proposed. First, the development of real-time preprocessing techniques specifically tailored for wearable EEG devices is essential. These algorithms should aim to minimize noise and artifacts caused by dry electrodes, such as eye movements, muscle activity, and external interference, to ensure higher fidelity in EEG recordings for reliable real-world applications. Second, while this study focused on resting-state EEG, further research should evaluate device performance across various cognitive tasks, including attention and memory assessments, mental workload evaluations [32], and brain–computer interface (BCI) experiments. This will provide valuable insights into their utility for applications like neurofeedback, cognitive state monitoring, and therapeutic interventions.

Third, a systematic investigation into the impact of dry-electrode technology on EEG signal quality is needed, particularly regarding impedance and signal stability over longer recording sessions, noise susceptibility in specific frequency bands like delta and theta, and comparisons with gel-based systems under both controlled and real-world conditions. Fourth, to enhance generalizability, future studies should include larger sample sizes with diverse demographics, considering specific subgroups such as older adults, individuals with neurological conditions, and participants with varying cognitive states like stress, fatigue, and meditation. Fifth, longitudinal studies are necessary to assess the test–retest reliability of wearable EEG devices, which will establish their consistency and applicability in long-term monitoring and training scenarios. Finally, comparative studies exploring emerging wearable EEG technologies are critical to evaluate their performance against established consumer-grade devices. These studies should focus on design constraints, artifact susceptibility, and signal accuracy across different platforms.

By addressing these key areas, future research can drive advancements in the design, signal processing, and practical application of wearable EEG devices, ultimately bridging the gap between research-grade systems and consumer-grade technologies.

## 6. Conclusions

In conclusion, it is essential for researchers and practitioners to consider the specific design capacities and limitations of individual devices as they impact the device’s ability to detect brain activity in various frequency bands. When developing neurotech products and user-friendly metrics, it is crucial for developers to address these limitations. Additionally, further evaluation and validation of the signals captured by consumer-grade EEG devices are necessary to prevent users from receiving misleading interpretations of brain activity.

- This study demonstrates that consumer-grade EEG devices vary in their ability to capture high-quality brain activity signals. Among the tested devices, the PSBD Headband exhibited the strongest alignment with research-grade equipment, while the PSBD Headphones demonstrated moderate performance and the Muse device showed the poorest performance. These results emphasize the importance of understanding the specific design limitations of consumer-grade EEG devices for researchers and consumers. The accuracy and sensitivity of these devices in detecting brain signals are limited compared to research-grade systems, particularly regarding noise levels and signal artifacts.
- This study underscores the need for ongoing research and development to improve the reliability of consumer EEG devices and enhance their signal processing algorithms to reduce artifacts. This is especially pertinent for devices like Muse, which exhibit significant differences in signal quality. While PSBD devices hold promise for applications such as neurofeedback training or cognitive state monitoring, users and developers should consider limitations in lower-frequency bands, especially during tasks that may involve eye movement or muscle tension.
- This study recommends that users should utilize such devices in controlled environments, particularly during calm periods with minimal eye or body movements, to reduce potential errors in signal interpretation, particularly in low-frequency bands like delta and theta rhythms.

Advantages, Disadvantages, and Recommendations

- The PSBD Headband Pro demonstrated strong alignment with the research-grade system, particularly in the occipital region, making it reliable for tasks such as alpha suppression during calm recording conditions. However, its susceptibility to low-frequency artifacts requires careful use. The PSBD Headphones Lite provided moderate signal quality, particularly for temporal EEG, but noise artifacts at specific frequencies limit its use in central EEG analysis. The Muse S Gen 2, despite its affordability and accessibility, displayed the poorest performance due to significant artifacts and poor temporal signal quality. As such, the Muse device is not recommended for high-fidelity EEG studies but may be used for basic frontal-region neurofeedback tasks.

These findings provide researchers and practitioners with a clear understanding of the strengths and weaknesses of each device. By addressing these limitations and following the outlined recommendations, future studies can better leverage consumer-grade EEG systems for specific applications while ensuring reliable and accurate results.

## Figures and Tables

**Table 1:** The summary of the devices’ montages and their mirror version in BP settings.

Device	Number of Electrodes	Ref.	GND	BP Mirror-Montage Specification
**PSBD Headband Pro**	4: T3, T4, O1, O2	FPz	FP1, FP2	BP-band Electrodes: O1, O2, T7 (~T3), T8 (~T4) Ref.: FPz GND: FP1
**Muse S** **Gen 2**	4: AF7, AF8, TP9, TP10	FPz	FP1, FP2	BP-band Electrodes: F7 (~AF7), F8 (~AF8), T7 (~TP9), T8 (~TP10) Ref.: FPz GND: FP1
**PSBD Headphones Lite**	4: C3, C4, A1, A2	Cz	FT9	BP-headphones Electrodes: C3, C4, TP9 (~A1), TP10 (~A2) Ref.: Cz GND: FT9

## Data Availability

The data presented in this study are available on request from the corresponding author due to the sensitivity of the project.
